# Recent advances in the use of extracellular vesicles from adipose-derived stem cells for regenerative medical therapeutics

**DOI:** 10.1186/s12951-024-02603-4

**Published:** 2024-06-06

**Authors:** Song Yang, Yiran Sun, Chenchen Yan

**Affiliations:** 1 Institute of Life Sciences, Chongqing Medical University, Chongqing, 400016 People’s Republic of China; 2 School of Pharmacy, Chengdu Medical College, Chengdu, 610500 People’s Republic of China

**Keywords:** Adipose-derived stem cell, Extracellular vesicle, Regenerative medicine, Tissue regeneration, Therapeutic effect, Underlying mechanism

## Abstract

Adipose-derived stem cells (ADSCs) are a subset of mesenchymal stem cells (MSCs) isolated from adipose tissue. They possess remarkable properties, including multipotency, self-renewal, and easy clinical availability. ADSCs are also capable of promoting tissue regeneration through the secretion of various cytokines, factors, and extracellular vesicles (EVs). ADSC-derived EVs (ADSC-EVs) act as intercellular signaling mediators that encapsulate a range of biomolecules. These EVs have been found to mediate the therapeutic activities of donor cells by promoting the proliferation and migration of effector cells, facilitating angiogenesis, modulating immunity, and performing other specific functions in different tissues. Compared to the donor cells themselves, ADSC-EVs offer advantages such as fewer safety concerns and more convenient transportation and storage for clinical application. As a result, these EVs have received significant attention as cell-free therapeutic agents with potential future application in regenerative medicine. In this review, we focus on recent research progress regarding regenerative medical use of ADSC-EVs across various medical conditions, including wound healing, chronic limb ischemia, angiogenesis, myocardial infarction, diabetic nephropathy, fat graft survival, bone regeneration, cartilage regeneration, tendinopathy and tendon healing, peripheral nerve regeneration, and acute lung injury, among others. We also discuss the underlying mechanisms responsible for inducing these therapeutic effects. We believe that deciphering the biological properties, therapeutic effects, and underlying mechanisms associated with ADSC-EVs will provide a foundation for developing a novel therapeutic approach in regenerative medicine.

**Graphical Abstract:**

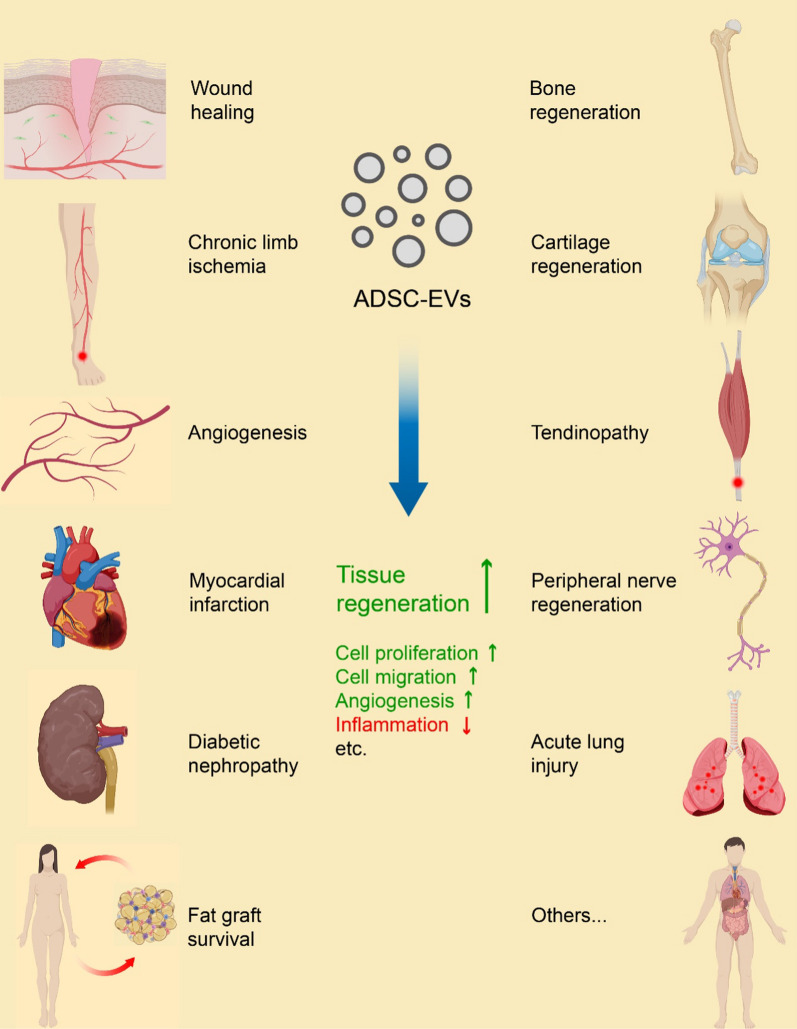

## Introduction

Adipose tissue, a highly versatile, dynamic and complex tissue [1–3], is now recognized as a vital endocrine organ that plays a crucial role in maintaining systemic metabolic homeostasis [4–6]. It primarily consists of mature adipocytes which make up the floating fraction, and the pelleted cellular components of the stromal vascular fraction (SVF), obtained through the conventional enzymatic isolation protocol [7, 8]. The SVF is widely acknowledged as a heterogeneous cell population composed of adipose-derived stem cells (ADSCs), preadipocytes, pericytes, endothelial precursor cells, endothelial cells, smooth muscle cells, fibroblasts, hematopoietic cells, macrophages and other immunocytes [7–9]. ADSCs are multipotent mesenchymal stem cells (MSCs) derived from the embryonic mesoderm. Although making up only about 12–18% of the total adipose cell population [10], ADSCs are the most regeneratively active cell fraction of adipose tissue, capable of renewing their population and differentiating into multiple cell lineages, such as adipocytes, myocytes, chondrocytes, and osteocytes [11, 12]. Due to their multipotency and robust proangiogenic, proepithelial, neurotrophic, antifibrotic, antiapoptotic, and immunomodulatory effects demonstrated in numerous in vitro and in vivo studies, ADSCs have been extensively utilized in the field of regenerative medicine and cell therapy [8, 11, 13, 14]. MSCs can be directly administered to the injured area in vivo, or a novel cell-free approach utilizing extracellular vesicles (EVs) can replace live stem cells. Because the therapeutic effect of MSCs primarily relies on the autocrine/paracrine action of growth factors, immunomodulators, cytokines, and other bioactive molecules secreted by the cells and enclosed within EVs [13, 14].

**Fig. 1:**
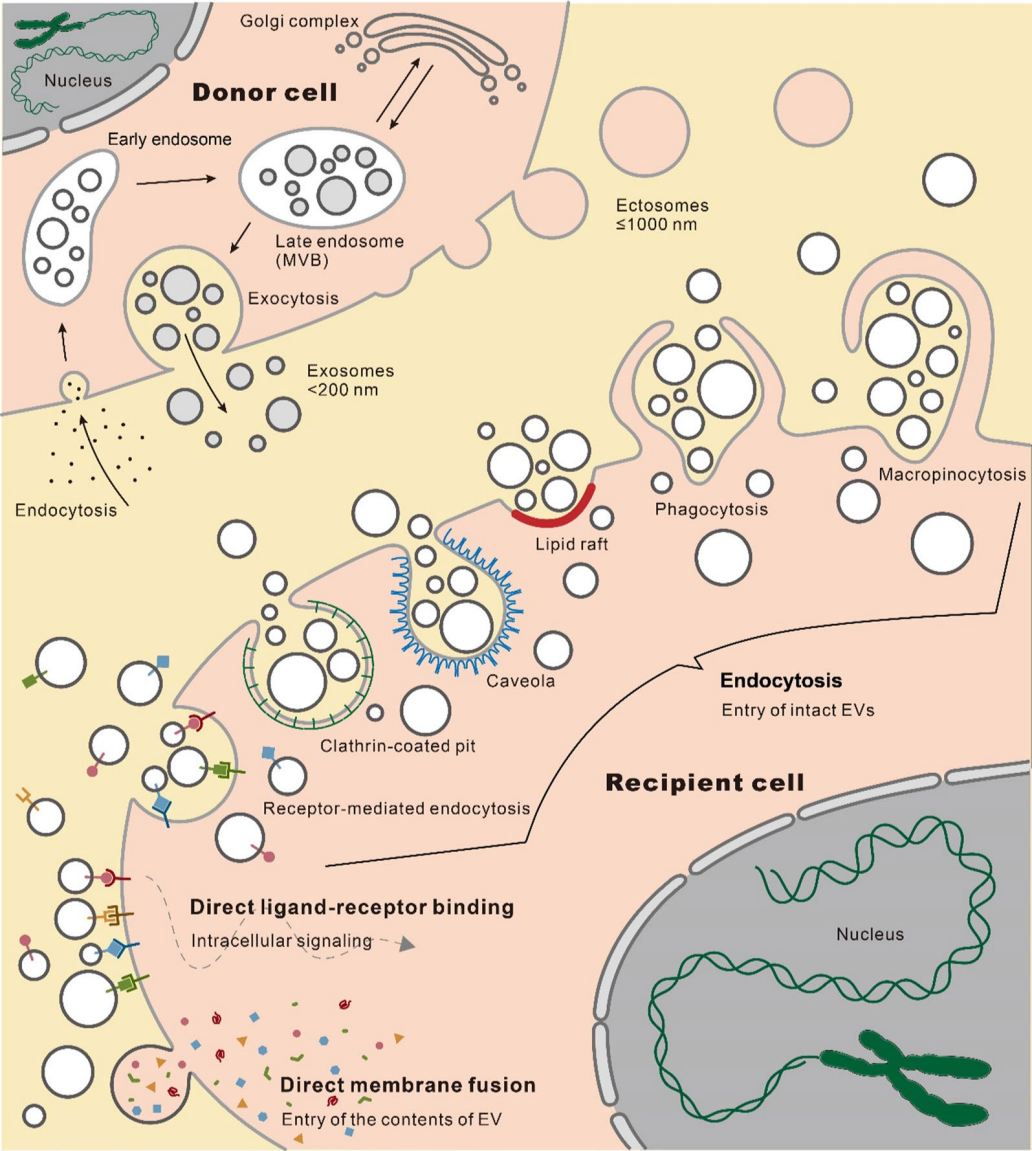
Cellular biogenesis and uptake of EVs. The EV biogenesis pathways include the outward budding of plasma membrane domains to form ectosomes, and the development of endosomes to release exosomes. The EV uptake pathways include entry of intact EVs through endocytosis, direct ligand-receptor binding triggered intracellular signaling without internalization or releasing the contents, and the release of EV contents inward via direct membrane fusion

EVs are nanosized bilayer lipid membrane structures secreted by cells that carry various biomolecules from donor cells, and cannot replicate on their own [15, 16]. These biomolecular cargos include lipids, proteins, chemical compounds, multimolecular complexes, DNA, RNA (siRNA, miRNA, lncRNA, circRNA, etc*.*), and even intact subcellular organelles [17–20]. Revealing the details of the molecular composition, diverse structures, and unique sequences of these EV cargos through nucleic acid sequencing or mass spectrometry provides valuable insights into the metabolic state of the parent cells and positions EVs as promising clinical biomarkers [21, 22]. EVs can be released by most cell types and have been detected in a variety of solid tissues and biofluids, such as blood, breast milk, saliva, urine, semen, bile, ascites, synovial fluid, cerebrospinal fluid, and amniotic fluid, making them suitable for intercellular signal transmission [17, 23–25]. The uptake of EVs by recipient cells initiates intercellular signaling, which forms the basis of their therapeutic potential [26–28]. This process can occur through different pathways: intact EVs entering recipient cells via endocytosis mediated by receptors, clathrin-coated pits, lipid rafts, caveolae, phagocytosis and macropinocytosis; direct ligand-receptor binding triggered intracellular signaling without internalization or content release; or the release of EV contents into the cytoplasm through direct membrane fusion (Fig. 1) [27]. EV-mediated intercellular signaling has been extensively investigated in most human diseases and related biological processes, including development [29], immunity [30–34], virus infection [35], tissue regeneration [36–39], obesity and diabetes mellitus [40–44], liver diseases [45–47], cardiovascular disorders [48–54], neurodegenerative diseases [55–60], aging [61–63], and cancer [64–69]. In addition, the potential utility of EVs as diagnostic biomarkers [70, 71] and cell-free curative agents [72, 73] for future clinical application has also been widely explored.

MSCs have been found to present in different tissues. Until now, the most employed human tissues as MSC-EV sources for therapeutic research and clinical trials include adipose tissue, bone marrow, and umbilical cord (Table 1) [74, 75]. Adipose tissue offers substantial advantages in its significant greater amount of clinical MSC source, resulting higher production of MSC-EVs, and the easier and less invasive surgical procedure to obtain the source tissue such as liposuction compared to other tissue sources [76, 77]. In addition, adipose-derived MSCs are reported to possess higher stability in culture conditions and lower senescence ratio by comparison [78]. Accumulating evidence suggests that ADSC-derived EVs (ADSC-EVs) possess cell therapy bioactivity similar to that of their parent cells while circumventing safety concerns associated with the administration of live stem cells [75, 79–81]. As a result, the number of registered clinical trials using ADSC-EVs as interventions is growing according to the ClinicalTrials.gov (Table 1, available online: http://www.clinicaltrials.gov/accessed on 23/Apr/2024). In this review, we present a concise overview of EV biogenesis and classification, with a primary emphasis on the latest advancements in tissue regenerative therapeutic potential, applications, benefits, and current limitations of ADSC-EVs in the field of regenerative medicine.

**Table 1:** Registered clinical trials with MSC-EV interventions on clinicaltrials.gov (http://www.clinicaltrials.gov/) accessed on 23/Apr/2024

NCT number	Study title	Conditions	Interventions	Phases	Last update
NCT04388982	The safety and the efficacy evaluation of allogenic adipose MSC-exos in patients with alzheimer's disease	Alzheimer disease	ADSC-EVs	Phase 1 | Phase 2	2021/6/25
NCT04276987	A pilot clinical study on inhalation of mesenchymal stem cells exosomes treating severe novel coronavirus pneumonia	Coronavirus	ADSC-EVs	Phase 1	2020/9/7
NCT04544215	A clinical study of mesenchymal progenitor cell exosomes nebulizer for the treatment of pulmonary infection	Drug-resistant	ADSC-EVs	Phase 1 | Phase 2	2020/9/10
NCT04313647	A tolerance clinical study on aerosol inhalation of mesenchymal stem cells exosomes in healthy volunteers	Healthy	ADSC-EVs	Phase 1	2021/8/4
NCT04270006	Evaluation of adipose derived stem cells exo.in treatment of periodontitis	Periodontitis	ADSC-EVs	EARLY_PHASE1	2020/2/17
NCT04657458	Expanded access for use of bmmsc-derived Extracellular Vesicles in Patients with COVID-19 associated ARDS	Covid19 | ARDS | hypoxia | cytokine storm	Bone marrow MSC-EVs (ExoFlo™)	N/A	2024/2/12
NCT05176366	Study of exoflo^™^ for the treatment of medically refractory ulcerative colitis	Ulcerative colitis | inflammatory bowel diseases	Bone marrow MSC-EVs (ExoFlo™)	Phase 1	2024/2/12
NCT05127122	Bone marrow mesenchymal stem cell derived extracellular vesicles infusion treatment for ARDS	ARDS, human	Bone marrow MSC-EVs (ExoFlo™)	Phase 1 | Phase 2	2024/2/12
NCT05354141	Extracellular vesicle treatment for acute respiratory distress syndrome (ARDS) (EXTINGUISH ARDS)	Acute respiratory distress syndrome | ARDS	Bone marrow MSC-EVs (ExoFlo™)	Phase 3	2024/2/12
NCT05125562	Extracellular vesicles infusion treatment for mild-to-moderate COVID-19	COVID-19	Bone marrow MSC-EVs (ExoFlo™)	Phase 2	2024/2/13
NCT04493242	Extracellular vesicle infusion treatment for COVID-19 associated ARDS	COVID-19 | ARDS	Bone marrow MSC-EVs (ExoFlo^™^)	Phase 2	2024/2/13
NCT05215288	Expanded access for use of exoflo^™^ in abdominal solid organ transplant patients	Solid organ transplant rejection | organ rejection transplants | organ rejection	Bone marrow MSC-EVs (ExoFlo^™^)	N/A	2024/2/12
NCT05130983	Study of ExoFlo^™^ for the treatment of medically refractory Crohn's disease	Crohn disease | inflammatory bowel diseases	Bone marrow MSC-EVs (ExoFlo™)	Phase 1	2024/2/12
NCT05116761	ExoFlo^™^ infusion for post-acute COVID-19 and chronic post-COVID-19 syndrome	Covid19 | postviral syndrome | dyspnea	Bone marrow MSC-EVs (ExoFlo^™^)	Phase 1 | Phase 2	2024/2/12
NCT05836883	Study of ExoFlo^™^ for the Treatment of Perianal Fistulas	Perianal Fistula | Crohn's Disease	Bone marrow MSC-EVs (ExoFlo^™^)	Phase 1 | Phase 2	2024/2/12
NCT04173650	MSC EVs in dystrophic epidermolysis bullosa	Dystrophic epidermolysis bullosa	Bone marrow MSC-EVs (AGLE 102)	Phase 1 | Phase 2	2024/4/4
NCT05078385	Safety of extracellular vesicles for burn wounds	Burns	Bone marrow MSC-EVs (AGLE 102)	Phase 1	2024/3/29
NCT03857841	A safety study of iv stem cell-derived extracellular vesicles (UNEX-42) in preterm neonates at high risk for BPD	Bronchopulmonary dysplasia	Bone marrow MSC-EVs (UNEX-42)	Phase 1	2021/10/12
NCT06242379	Safety and efficacy of stem cell small extracellular vesicles in patients with retinitis pigmentosa	Retinitis pigmentosa	Bone marrow MSC-EVs	Phase 1 | Phase 2	2024/3/27
NCT05808400	Safety and efficacy of umbilical cord mesenchymal stem cell exosomes in treating chronic cough after COVID-19	Long COVID-19 syndrome	Umbilical cord MSC-EVs	EARLY_PHASE1	2023/4/14
NCT05787288	A clinical study on safety and effectiveness of mesenchymal stem cell exosomes for the treatment of COVID-19	COVID-19 pneumonia	Umbilical cord MSC-EVs	EARLY_PHASE1	2023/4/7
NCT02138331	Effect of microvesicles and exosomes therapy on β-cell mass in type i diabetes mellitus (T1DM)	Diabetes mellitus type 1	Umbilical cord MSC-EVs	Phase 2 | Phase 3	2014/5/14
NCT05871463	Effect of mesenchymal stem cells-derived exosomes in decompensated liver cirrhosis	Decompensated liver cirrhosis	Umbilical cord MSC-EVs	Phase 2	2023/5/23
NCT04213248	Effect of UMSCs derived exosomes on dry eye in patients with cGVHD	Dry eye	Umbilical cord MSC-EVs	Phase 1 | Phase 2	2022/2/11
NCT03437759	MSC-exos promote healing of MHs	Macular holes	Umbilical cord MSC-EVs	EARLY_PHASE1	2021/4/6
NCT04356300	Exosome of mesenchymal stem cells for multiple organ dysfuntion syndrome after surgical repair of acute type a aortic dissection	Multiple organ failure	Umbilical cord MSC-EVs	N/A	2020/5/6
NCT06245746	UCMSC-exo for chemotherapy-induced myelosuppression in acute myeloid leukemia	Acute myeloid leukemia | neutropenia | anemia | thrombocytopenia | infections | bleeding	Umbilical cord MSC-EVs	Phase 1	2024/2/9
NCT05669144	Co-transplantation of mesenchymal stem cell derived exosomes and autologous mitochondria for patients candidate for CABG surgery	Myocardial infarction | myocardial ischemia | myocardial stunning	Umbilical cord MSC-EVs and autologous mitochondria	Phase 1 | Phase 2	2022/12/30
NCT05413148	The effect of stem cells and stem cell exosomes on visual functions in patients with retinitis pigmentosa	Retinitis pigmentosa	Umbilical cord (wharton jelly) MSCs | umbilical cord (wharton jelly) MSC-EVs	Phase 2 | Phase 3	2022/9/7
NCT05387278	Safety and effectiveness of placental derived exosomes and umbilical cord mesenchymal stem cells in moderate to severe acute respiratory distress syndrome (ARDS) associated with the novel corona virus infection (COVID-19)	COVID-19 acute respiratory distress syndrome | respiratory distress syndrome	Placenta/umbilical cord MSC-EVs (EV-Pure^™^ and WJ-Pure^™^)	Phase 1	2022/10/13
NCT06072794	A proof of concept study to evaluate exosomes from human mesenchymal stem cells in women with premature ovarian insufficiency (POI)	Premature ovarian insufficiency | diminished ovarian reserve	Placenta MSC-EVs (VL-PX10)	Phase1	2024/1/3
NCT05402748	Safety and efficacy of injection of human placenta mesenchymal stem cells derived exosomes for treatment of complex anal fistula	Fistula perianal	Placenta MSC-EVs	Phase 1 | Phase 2	2022/11/2
NCT05658094	Exosome effect on prevention of hairloss	Hair loss | alopecia	Placenta MSC-EVs	N/A	2022/12/20
NCT05499156	Safety of injection of placental mesenchymal stem cell derived exosomes for treatment of resistant perianal fistula in Crohn's patients	Perianal fistula in patients with Crohn's disease	Placenta MSC-EVs	Phase 1 | Phase 2	2022/8/12
NCT05261360	Clinical efficacy of exosome in degenerative meniscal injury	Knee; injury, meniscus (lateral) (medial) | meniscus tear | meniscus lesion | meniscus; degeneration | meniscus; laceration | meniscus injury, tibial | knee injuries | knee pain swelling | arthralgia	Synovial fluid MSCs | synovial fluid MSC-EVs	Phase 2	2022/3/4
NCT05738629	Safety and efficacy of pluripotent stem cell-derived mesenchymal stem cell exosome (PSC-MSC-Exo) eye drops treatment for dry eye diseases post refractive surgery and associated with blepharospasm	Dry eye disease	Pluripotent stem cell MSC-EVs	Phase 1 | Phase 2	2023/2/22
NCT05881668	MSC-EV in acute-on-chronic liver failure after liver transplantation	Liver failure, acute on chronic	MSC-EVs (tissue source not provided)	Phase 1	2023/10/12
NCT05940610	The safety and efficacy of MSC-EVs in acute/acute-on-chronic liver failure	Acute-on-chronic liver failure | acute liver failure	MSC-EVs (tissue source not provided)	Phase 1 | Phase 2	2023/10/12
NCT05523011	Safety and tolerability study of msc exosome ointment	Psoriasis	MSC-EVs (tissue source not provided)	Phase 1	2022/8/31
NCT04798716	The use of exosomes for the treatment of acute respiratory distress syndrome or novel coronavirus pneumonia caused by COVID-19	Covid19 | novel coronavirus pneumonia | acute respiratory distress syndrome	MSC-EVs (tissue source not provided)	Phase 1 | Phase 2	2022/3/11
NCT04602104	A clinical study of mesenchymal stem cell exosomes nebulizer for the treatment of ARDS	Acute respiratory distress syndrome	MSC-EVs (tissue source not provided)	Phase 1 | Phase 2	2021/11/2
NCT04850469	Study of MSC-exo on the therapy for intensively ill children	Sepsis | critical illness	MSC-EVs (tissue source not provided)	N/A	2022/6/15
NCT05191381	Immune modulation by exosomes in COVID-19	COVID-19 | critical illness | hypercytokinemia | lung fibrosis	MSC-EVs (tissue source not provided)	N/A	2024/1/17
NCT05813379	Mesenchymal stem cells derived exosomes in skin rejuvenation	Anti-aging	MSC-EVs (tissue source not provided)	Phase 1 | Phase 2	2023/4/14
NCT03384433	Allogenic mesenchymal stem cell derived exosome in patients with acute ischemic stroke	Cerebrovascular disorders	MSC-EVs (tissue source not provided)	Phase 1 | Phase 2	2021/1/25
NCT05216562	Efficacy and safety of EXOSOME-MSC therapy to reduce hyper-inflammation in moderate COVID-19 patients	SARS-CoV2 infection	MSC-EVs (tissue source not provided) | COVID-19 standard treatment	Phase 2 | Phase 3	2022/2/18

## Classification and biogenesis of EVs

Although the classification of EVs may vary from time to time, the prevailing categorization is based on the biogenesis and size of vesicles, resulting in two major classes: ectosomes and exosomes [27, 82]. Ectosomes or microvesicles are characterized by their direct budding outward from the plasma membrane, with a diameter ranging from approximately 50 to 1000 nm (Fig. 1) [15, 16, 27]. The sprouting of the cell membrane is triggered by phospholipid redistribution within the bilayer, which is initiated by activation of the phospholipid crawling enzyme and facilitated by calcium-dependent degradation of the cytoskeleton that is bound to the membrane [23]. The arrestin domain-containing protein 1 (ARRDC1) plays a key role in driving cell membrane sprouting by acting as an anchor fusion molecule that facilitates the sorting of specific macromolecules into microvesicles [23, 83, 84]. ARRDC1 recruits endosomal sorting complex required for transport (ESCRT) proteins, including tumor susceptibility gene 101 (TSG101) and vacuolar protein sorting-associated protein 4 (VPS4), to the plasma membrane to promote the budding process [23, 83, 84].

In contrast, exosomes originate from endosomes and multivesicular bodies (MVBs) and are approximately 40–200 nm in diameter [16, 27, 85]. The whole formation process of exosomes is highly intricate: initially, inward budding endocytosis gives rise to early endosomes, which subsequently mature into late endosomes containing intraluminal vesicles that accumulate in the lumen under communication with the Golgi complex. Eventually, these late endosomes transform into MVBs that fuse with the cell membrane and release their contents, the intraluminal vesicles, into the extracellular space as exosomes (Fig. 1) [27, 82, 86]. Recent reports suggest that both ESCRT-dependent and ESCRT-independent sorting pathways are involved in loading MVBs with cargo during maturation [23].

Besides the two major and well-studied categories, EVs could be released through other cellular processes with different average sizes. For example, disassembly of apoptotic cells gives rise to the apoptotic body with a diameter of 800–5000 nm, which contains components and information from the dying cells and are able to deliver these to healthy recipient cells [87]. The newly discovered migrasome, with a size of 500–3000 nm, plays an important role in fields such as intercellular communication, homeostasis maintenance, embryonic development, and disease progression [88]. Their formation depends on cell migration and is closely associated with tetraspanins (TSPANs) and integrins [88]. And, the recently identified “large oncosome” has an atypical large size of 1–10 μm [89]. They are specifically originated through oncocyte plasma membrane budding with oncogenic materials carried inside, and might share biogenesis pathways with ectosomes [89].

Unfortunately, most EV isolation procedures do not separate EVs by their biogenesis mechanisms, and universal definitive molecular markers of these EV subtypes are missing. Therefore, In this review, we use the term “extracellular vesicle” to encompass various types of secreted vesicular structures as recommended [16].

## Regenerative therapeutic potential of ADSC-EVs

The aforedescribed biogenesis pathways of EV load them with diverse biomolecules derived from the donor cells. As a result, these EVs mimic the function of their donor cells by initiating target signaling pathways in the recipient cells with their cargos. Substantial studies have aimed to elucidate the ADSC-EV-enclosed cargos, their respective molecular targets and the activated signaling cascades in the recipient cells, and the resulting functions within the specific context of medical conditions or diseases (Table 2). This knowledge serves as a prerequisite for designing innovative therapeutic strategies based on ADSC-EVs.

**Table 2:** Molecular machinery responsible for ADSC-EV-driven regenerative therapeutic effects

ADSC-EV cargo	Target/signaling	Conditions/diseases	Action	Ref
miR-126-3p	PIK3R2/PTEN/PI3K/AKT	Acute wounds	Fibroblast proliferation and migration↑, HUVEC angiogenesis↑, wound healing↑, collage deposition↑, tube formation↑	[90]
miR-125a-3p	PTEN	Acute wounds	Wound healing↑, HUVEC viability↑, migration↑, angiogenesis↑	[91]
miR-21	TGF-β1/PI3K/AKT	Acute wounds	HaCaT cell proliferation and migration↑	[92]
miR-19b	CCL1/TGF-β	Acute wounds	HaCaT cell damage↓, Inflammation↓, apoptosis↓	[93]
miR-486-5p	Sp5/cyclin D2	Acute wounds (scars)	Re-epithelialization↑, collagen synthesis↑, angiogenesis↑, scar formation↓	[94]
miR-192-5p	IL-17RA/SMAD	Acute wounds (scars)	HSF proliferation and migration↓, collagen deposition↓, hypertrophic scar fibrosis↓, wound healing↑	[95]
miR-132	NF-κB	Chronic diabetic wounds	Diabetic wound healing↑, collagen deposition↑, ECM fibronectin accretion↑, angiogenesis↑, M2 polarization↑, inflammation↓, HUVEC proliferation and migration↑	[96]
miR-210	RUNX3	Chronic limb ischemia	HUVEC proliferation and migration↑, invasion↑	[97]
miR-126	SPRED1/ERK1/2	Angiogenesis	Endothelial cell proliferation and migration↑, angiogenesis↑	[98]
miR-125a	DLL4/Notch1	Angiogenesis	HUVEC angiogenesis↑	[99]
miR-423-5p	SUFU	Angiogenesis	HUVEC proliferation and migration↑, angiogenesis↑	[100]
miR-31	FIH1/HIF-1α	Angiogenesis | myocardial infarction	HUVEC migration↑, tube formation↑, angiogenesis↑	[101, 102]
miR-486	SMAD1/mTOR	Diabetic nephropathy	MP5 cell autophagy↑, cell viability↑, apoptosis↓	[103]
miR-26a-5p, miR-147-3p	TLR4/NF-κB	Diabetic nephropathy | Achilles tendinopathy	MP5 cell viability↑, apoptosis↓; tendon cell proliferation↑, collagen production↑, Inflammation↓, M1 polarization↓, M2 polarization↑	[104, 105]
miR-375	IGFBP3	Bone regeneration	Bone regeneration↑, human BMSC osteogenic differentiation↑	[106]
miR-21-5p	ACVR2A/SMAD2/3, BTG2/IRS1/AKT	Bone regeneration | polycystic ovary syndrome	Bone loss↓, osteoclastogenesis↓; ovarian polycystin↓, fertility↑, metabolic disturbances↓	[107, 108]
miR-26b	KPNA2	Peripheral nerve regeneration	Schwann cell autophagy↓, myelin sheath regeneration↑	[109]
miR-22-3p	PTEN/AKT/mTOR	Peripheral nerve regeneration	Schwann cell proliferation and migration↑, dorsal root ganglion growth↑	[110]
miR-125b-5p	KEAP1/NRF2/GPX4	Acute lung injury	Lung injury↓, death rate↓, oxidative stress injury↓, lung tissue ferroptosis↓, PMVEC inflammation↓, ROS↓, cell injury↓, cell ferroptosis↓	[111]
miR-150-5p	CXCL1	Hepatic fibrosis	Hepatic fibrosis↓, collagen volume fraction↓, inflammation↓, hepatic injury↓, hepatic stellate cell proliferation↓, activation↓	[112]
lncRNA H19	miR-19b/SOX9/Wnt/β-catenin	Acute wounds	HDF proliferation and migration↑, invasion↑	[113]
lncRNA MALAT1	miR-124/Wnt/β-catenin	Acute wounds	Cutaneous wound healing↑, HaCaT and HDF proliferation↑, migration↑, apoptosis↓	[114]
linc00511	PAQR3/TWIST1	Chronic diabetic wounds	DFU healing↑, Endothelial progenitor cell proliferation and migration↑, angiogenesis↑	[115]
circ-Snhg11	miR-144-3p/NRF2/ARE/HIF-1α	Chronic diabetic wounds	Endothelial cell damage↓, endothelial progenitor cell dysfunction↓, M1-like polarization↓, ROS↓, M2-like polarization↑, diabetic wound healing↑, endothelial cell proliferation and migration↑, angiogenesis↑	[116, 117]
circ-Gcap14	miR-18a-5p/HIF-1α/VEGF	Chronic diabetic wounds	Diabetic wound healing↑, angiogenesis↑	[118]
mmu_circ_0001052	miR-106a-5p/FGF4/p38MAPK	Chronic diabetic wounds	DFU wound healing↑, high glucose-treated HUVEC apoptosis↓, proliferation, migration and angiogenesis↑	[119]
mmu_circ_0000250	miR-128-3p/SIRT1	Chronic diabetic wounds	Diabetic wound healing↑, angiogenesis↑, high glucose-treated endothelial progenitor cell dysfunction↓, autophagy↑, apoptosis↓	[120]
HSP90	LRP1/AKT	Chronic diabetic wounds	Diabetic wound healing↑, collagen deposition↑, neovascularization↑, keratinocyte, fibroblast and endothelial cell proliferation and migration↑, angiogenesis↑, ROS↓, apoptosis↓	[121]
HIF-1α	Multiple genes of growth factors	Chronic diabetic wounds	Diabetic wound healing rate and quality↑, fibroblast proliferation and migration↑	[122]
NRF2	NRF2/ARE/HIF-1α	Chronic diabetic wounds	Diabetic wound healing↑, angiogenesis↑, inflammation↓, high glucose-treated human endothelial progenitor cell proliferation↑, ROS↓	[123]
IRF1	miR-16-5p/Sp5	Chronic diabetic wounds	Diabetic wound healing↑, fibroblast proliferation and migration↑, endothelial cell angiogenesis↑	[124]
GLO-1	Methylglyoxal/eNOS/AKT/ERK/P-38, Methylglyoxal/AP-1/ROS/NLRP3/ASC/Caspase-1/IL-1β	Chronic limb ischemia	Diabetic angiogenesis↑, blood perfusion↑, muscle structural integrity↑, high glucose-treated HUVEC proliferation and migration↑, tube formation↑, apoptosis↓	[125]
NRG1	EGFR/p38MAPK	Chronic limb ischemia | angiogenesis	Skeletal muscle regeneration and protection↑, angiogenesis↑, muscle function↑, myoblast proliferation↑, differentiation↑, apoptosis↓	[126]
OPG	RANKL	Bone regeneration	Bone loss↓, osteoclastogenesis↓	[107]
NAMPT	SIRT1/PPARγ/PGC1-α, SIRT1/NF-κB p65/NLRP3	Achilles tendinopathy	Tendinopathy↓, tenocyte viability↑, cell senescence↓, macrophage phagocytosis↑, M2 polarization↑	[127]
Mitochondrial component	mtDNA | mitochondrial membrane potential | OXPHOS | ATP generation	Acute lung injury	LPS-challenged Alveolar macrophage mitochondrial ROS stress↓, M2 polarization↑	[19]

## Wound healing

### Acute wounds

**Fig. 2:**
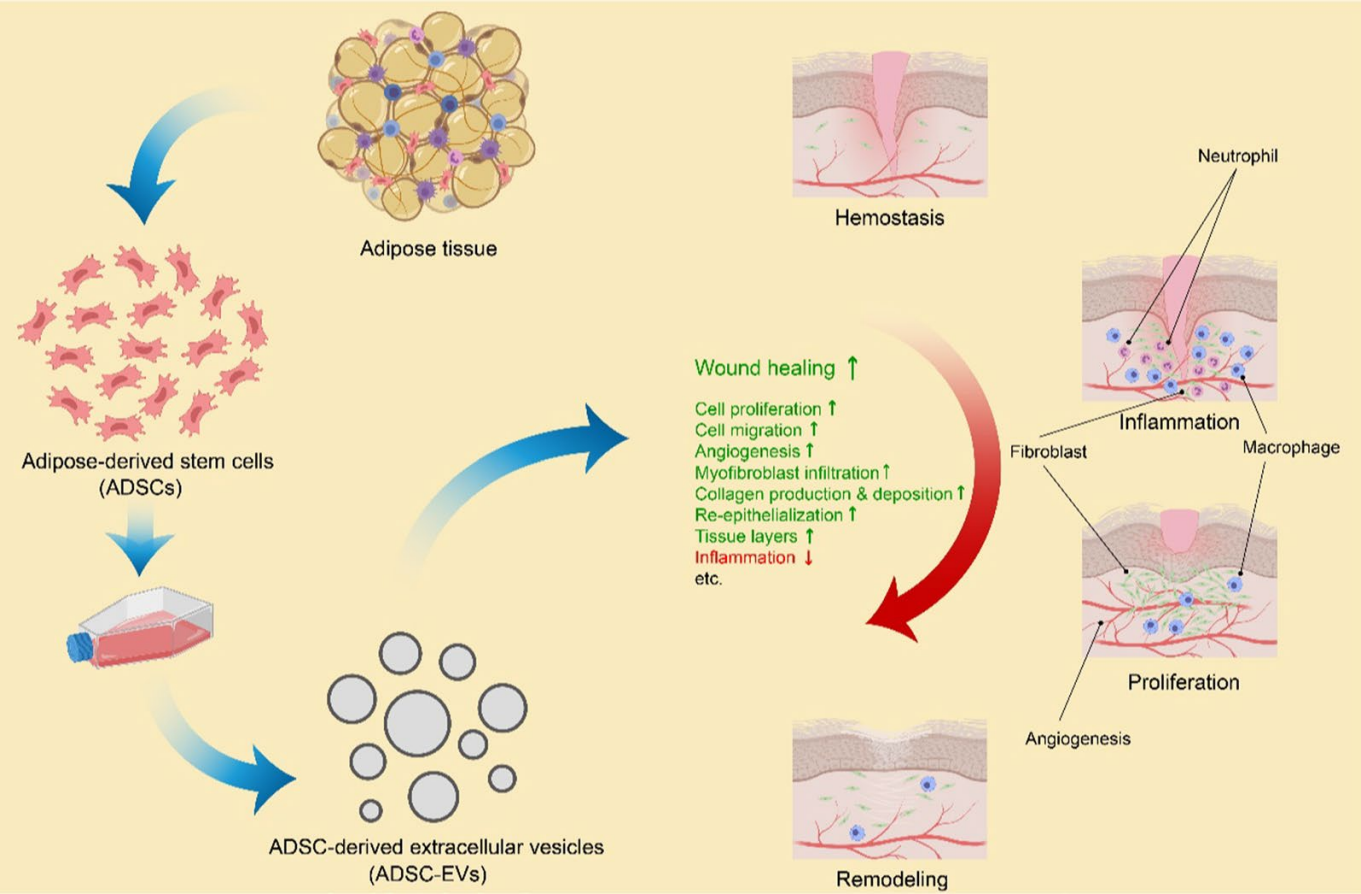
Preparation of ADSC-EVs, the four sequential phases of wound healing, and ADSC-EV-induced promotion of wound healing. ADSC-EVs promote wound healing by enhancing cell proliferation and migration, stimulating angiogenesis, facilitating myofibroblast infiltration, promoting collagen production and deposition, accelerating re-epithelialization and tissue layer growth, as well as suppressing inflammation when it is time to move on to the next phase. Created with MedPeer (medpeer.cn)

The wound healing of skin is one of the most extensively investigated regenerative medical conditions that employs ADSC-EVs as a therapeutic intervention, showing highly promising curative outcomes according to reported findings (Table 2) [76, 128, 129]. The skin, an innate protective shield against the external environment, is constantly exposed to potential injuries, making wound healing a vital process for the survival of all higher organisms [130]. As a process that is conserved throughout evolution across species, acute wound healing occurs in four sequential and overlapping phases [130–132] (Fig. 2). Hemostasis begins first when blood clotting occurs to control blood loss and prevent microbial invasion. This process is followed by inflammation, which overlaps with hemostasis and involves the recruitment of proinflammatory immunocytes, such as neutrophils (initially) and then macrophages, to remove tissue debris and foreign pathogens at the injury site. Activated macrophages release a variety of growth factors and cytokines at the wound site to amplify earlier signals, thereby playing a crucial role in the healing process. Subsequently, the proliferation phase begins concurrently with inflammation, which is characterized by angiogenesis, re-epithelialization, and fibroplasia to restore the lost tissue. Fibroblasts play a pivotal role in this phase by producing important factors, including collagen, elastin, and extracellular matrix (ECM) proteins. Finally, remodeling occurs simultaneously with tissue formation through processes involving cell maturation, cell apoptosis, ECM contraction resulting from the transition of fibroblasts to myofibroblasts, and the conversion of type III collagen to type I collagen through the coordinated actions of matrix metalloproteinases (MMPs) and tissue inhibitors of metalloproteinases (TIMPs). The remodeling phase may last from weeks to years until the desired new tissue is ultimately regenerated [131–134] (Fig. 2).

To date, ADSCs have emerged as a promising approach for skin regeneration and wound healing owing to their remarkable abundance and accessibility compared to MSCs from other tissue sources [135, 136]. ADSC-EVs have been shown to mimic the therapeutic activity of ADSCs while offering advantages such as greater stability, easier storage, and fewer safety and ethical concerns [128]. Extensive evidence supports the role of ADSC-EVs in accelerating tissue regeneration and suppressing inflammation when it is time to move on to the next phase of wound healing at the site of injury [137] (Fig. 2). In vitro studies have demonstrated that ADSC-EVs promote cell proliferation, migration, angiogenesis, and collagen production in human dermal fibroblasts (HDFs), human umbilical vein endothelial cells (HUVECs), and human epidermal keratinocytes (HaCaT cells) through regulating signaling pathways that control these processes [113, 138, 139]. Note that in a study, the ADSC-EV-treated HDF proliferation shows an average of a 120% increase compared to the control [138]. Animal experiments consistently exhibit improved healing efficiency characterized by increased re-epithelialization, thicker tissue layers, elevated vascularization, myofibroblast infiltration, and deposition of type III collagen, consequently leading to accelerated acute wound healing [113, 138, 139]. Despite the high variability, the ADSC-EVs display an approximately 10% increase in wound closure rate compared to the control [138]. Mechanistic studies have revealed the crucial roles of the Wnt/β-catenin, the phosphatidylinositol 3-kinase (PI3K)/AKT, and the extracellular signal-regulated kinase (ERK) signaling pathways, which are canonical regulators of stem cell potency, cell proliferation, survival, and growth, in mediating the ADSC-EV-driven healing process across multiple dermal cell lines [113, 139, 140]. In addition to their efficacy in excisional and incisional wounds, ADSC-EVs have shown potential in mitigating ultraviolet B (UVB)-induced skin photoaging [141]. In vivo administration of ADSC-EVs reduces skin wrinkles caused by UVB-induced photoaging in mice while increasing epidermal cell proliferation and decreasing macrophage infiltration and reactive oxygen species (ROS) levels. In vitro studies also reveal increased levels of antioxidant enzymes and decreased production of intracellular ROS in HDFs after ADSC-EV administration. This treatment enhances HDF activity, protects HDFs from UVB-induced senescence, induces HDF cell cycle arrest, and attenuates M1 macrophage polarization of RAW 264.7 cells [141]. Furthermore, ADSC-EVs have shown potential in the regeneration of skin appendages such as hair follicles, exhibiting validated phenotypes and the gene expression profile that supports the outcomes [142]. In summary, the multifaceted effects of ADSC-EVs on skin tissue repair and regeneration, similar to those of their donor cells, have been extensively acknowledged, and ongoing efforts are being made to develop optimal clinical therapies based on these effects [136, 143, 144].

Given the globally acknowledged therapeutic potential of ADSC-EVs in acute wound healing, researchers have been actively exploring strategies to enhance their bioactivity, reduce dosage frequency, and optimize clinical practice. Compared with negative control ADSC-EVs, miR-126-3p-overexpressing ADSC-EVs exhibit enhanced potential for the proliferation and migration of HDFs as well as angiogenesis in HUVECs [90]. Conversely, inhibition of either ADSC-EVs or miR-126-3p or phosphoinositide-3-kinase regulatory subunit 2 (PIK3R2), which is a regulatory subunit of PI3K and the intracellular target of miR-126-3p, results in the opposite effects. The potential of miR-126-3p-overexpressing ADSC-EVs is further verified through improvements in the wound healing rate, increased collagen deposition, and enhanced angiogenesis in a rat model [90]. In addition to bioengineering strategies, small molecules has been employed to enhance the therapeutic potential of ADSC-EVs [145]. Compared with control EVs from normal ADSCs, EVs derived from selenium-treated ADSCs lead to increased cell proliferation, migration, angiogenesis, and inflammatory suppression both in vitro and in vivo [145]. However, further investigation is needed to elucidate the underlying mechanism responsible for such improvements. Apart from their tunable therapeutic potential, as an excreted agent, EVs are rapidly eliminated by the circulation system, thus showing limited duration of efficacy at specific sites for tissue repair. Hydrogels are highly porous and loosely structured synthesized materials with excellent biocompatibility, making them an ideal choice as carriers for EVs to achieve a controlled release rate and prolonged retention time when needed [146]. For instance, the administration of Pluronic F-127 hydrogel-encapsulated human ADSC-EVs results in a reduction in dosage frequency without compromising therapeutic efficacy [147]. Compared to either subcomponent alone, the hydrogel/ADSC-EV complex enhances healing efficacy by promoting skin wound healing and re-epithelialization, stimulating collagen production, and elevating the expression of Ki67, α-smooth muscle actin (α-SMA), CD31, and skin barrier proteins while suppressing the expression of inflammatory factors [147]. To enhance the biocompatibility, a separate group of researchers employ a three-dimensional scaffold composed solely of type I collagen and platelet-rich plasma [148]. This scaffold provides natural support for cell adhesion, migration, and proliferation of keratinocytes and fibroblasts and serves as a carrier and release controller for EVs. Notably, their data demonstrate that the combination of ADSC-EVs and the scaffold effectively reduces inflammation levels and promotes cell proliferation and angiogenesis, ultimately leading to accelerated wound healing in a mouse model of full-thickness skin defects [148]. These findings have been further validated through proteomic analysis. In general, researchers’ endeavors to enhance the therapeutic efficacy and mitigate the adverse effects of ADSC-EVs through engineering approaches will significantly contribute to their global clinical application.

Skin wound healing commonly leads to scar formation, which is characterized by the absence of skin appendages and incomplete restoration of skin functionality (approximately 80% of intact skin strength), thereby causing significant aesthetic and psychological concerns [130, 132, 149]. The aberrant remodeling of the ECM and excessive collagen deposition are recognized as immediate factors contributing to scar development [149, 150]. Recent studies have indicated that ADSC-EVs play a supportive role in facilitating scarless cutaneous repair. Intravenous injection of ADSC-EVs has been shown to reduce scar size, suppress fibroblast differentiation into myofibroblasts, and increase the ratio of collagen III to collagen I, as well as the ratio of transforming growth factor-β3 (TGF-β3) to TGF-β1, in a mouse incisional wound model [151]. Furthermore, in vitro experiments on HDFs reveal that ADSC-EVs promote MMP3 expression and increase the MMP3/TIMP1 ratio by activating the ERK pathway, thereby modulating ECM remodeling [151]. Considering that myofibroblasts are the primary cell type responsible for ECM accumulation, with TGF-β signaling being a key pathway involved [150] and MMPs serving as major endopeptidases responsible for ECM degradation [152], it has been demonstrated that ADSC-EVs effectively mitigate scar formation by modulating ECM remodeling, as anticipated [151]. Moreover, scars can progress pathologically into hypertrophic scars and keloids characterized by uncontrolled fibroproliferation, the continuous accumulation of ECM and cells, and, in the case of keloids, invasion of adjacent healthy skin [150, 153, 154]. Fortunately, ADSC-EVs have also shown efficacy under both conditions. Treatment with ADSC-EVs results in reduced cell proliferation and migration in hypertrophic scar fibroblasts (HSFs), along with decreased expression of collagen proteins, interleukin-17 receptor A (IL-17RA), phosphorylated small mothers against decapentaplegic protein 2/3 (p-SMAD2/3), and increased levels of SMAD interacting protein-1 (SIP1) [95]. These effects are consistent with the accelerated wound healing and reduced hypertrophic collagen deposition observed in a mouse model. The mechanism underlying the effects of ADSC-EVs has been verified to involve the miR-192-5p/IL-17RA/SMAD axis through both overexpression and knockdown of IL-17RA [95]. In the case of keloids, ADSC-EVs have been shown to suppress collagen production and disrupt angiogenesis in keloid tissue explants [155]. In vitro tests utilizing keloid fibroblasts (KFs) demonstrate that the administration of ADSC-EVs effectively suppresses the expression of collagen I, collagen III, fibronectin, and α-SMA, as well as subsequent ECM production [155]. The leading molecular mechanisms underlying these effects are believed to involve the downregulation of the Notch 1 and TGF-β2/SMAD3 signaling pathways, concomitant with the upregulation of TGF-β3. Despite the increasing number of reports on ADSC-EV-induced attenuation of scar formation, further comprehensive investigations are imperative to elucidate the underlying mechanisms by which ADSC-EVs facilitate wound healing while inhibiting scar formation. Note that scar formation refers to the overprogression of healing process out of its physiological range, particularly the subprocesses of inflammation, cell proliferation and collagen deposition.

### Chronic diabetic wounds

The fast-growing prevalence of type 2 diabetes (T2D) in recent decades has rendered this disease a formidable global health threat, afflicting approximately 537 million adults (estimated 6.7 million deaths) in 2021 and projected to increase to around 643 million by 2030 [156, 157]. The current surge entails enormous economic loss due to combined medical expenses and diminished work productivity [158]. Moreover, the occurrence of comorbidities is common in T2D patients and contributes substantially to the disease burden [159]. Taking diabetic wounds as an example, it is estimated that approximately 20% to more than 30% of patients with diabetes will develop chronic nonhealing wounds, such as diabetic foot ulcers (DFUs), during their lifespan. These wounds exhibit considerable recurrence rates of up to 40% within one year and 65% within five years, with no reliable predictive methods currently available [158]. Additionally, more than 70% of DFU patients will ultimately require lower limb amputation, imposing a heavy financial burden and severe impairment to patients’ quality of life [160].

Diabetes impedes the healing process by disrupting each phase of wound healing mentioned earlier (Fig. 2). The intricate underlying etiology involves hyperglycemia, hemoglobin glycation, impaired neutrophil function, dysregulated macrophage polarization and function, sustained production of proinflammatory cytokines, impaired angiogenesis, reduced fibroblast and keratinocyte proliferation and migration, compromised growth factor production, decreased cytokine production, downregulated MMPs production, neuropathy, and nitrous oxide blockade [133, 158, 160]. These factors orchestrate the delay of healing process, thereby making diabetes a typical condition of chronic wounds, which generally fail to progress through the normal healing process and show no signs of healing in four weeks [131, 133]. In contrast to the acute wounds described above, which typically show signs of healing in less than four weeks following the normal progression of wound healing, chronic wounds are generally characterized by elevated bacterial levels caused by hyperglycemia along with high inflammatory cytokine levels, increased protease and ROS levels, a degraded nonfunctional ECM, reduced mitogenic activity, and enhanced cell senescence [131, 132, 158]. Despite these challenges posed by the complex pathology of chronic diabetic wounds, accumulating evidence suggests that ADSC-EVs have the potential to improve chronic diabetic wound healing through mechanisms similar to those in acute wounds [157].

The effects of ADSC-EVs on chronic diabetic wounds include facilitating cell proliferation and migration, inhibiting apoptosis, enhancing re-epithelialization and angiogenesis, reducing ROS levels, and suppressing inflammation, as evidenced by recent studies [116, 121, 161–164] (Fig. 2). Multiple molecular signaling pathways responsible for these beneficial effects have been identified since ADSC-EVs are essentially a heterogeneous collection of diverse biomolecules encapsulated within a lipid membrane that can activate versatile signaling cascades. Moreover, different research groups may focus on distinct aspects of this treatment. The depicted signaling pathways include: the PI3K/AKT pathway as a key regulator of cell proliferation and survival [121, 164], the Fas/Fas ligand (FasL) pathway as an initiator of cell death [161], the sirtuin 3 (SIRT3)/superoxide dismutase 2 (SOD2) axis as a scavenger of ROS [162], the TGF-β1/SMAD3 pathway as a crucial mediator of tissue repair and immune response [163], and the hypoxia-inducible factor-1α (HIF-1α) as a key factor in angiogenesis and immunomodulation [116]. These ongoing efforts to explore the efficacy and molecular mechanisms underlying ADSC-EV treatment for chronic diabetic wounds will serve as the cornerstone for potential medical intervention against this intractable condition.

Similar to acute wounds, bioengineered ADSC-EVs have gained popularity and reliability in addressing refractory chronic diabetic wounds with enhanced efficacy. Compared with control EVs, HIF-1α-overexpressing ADSC-EVs improve the wound healing rate and quality in a diabetic nude mouse model [122]. In vitro experiments in HDFs demonstrate increased expression of multiple growth factors and collagen proteins, which appears to be induced by the upregulation of PI3K/AKT pathway, which is crucial for cell proliferation and survival [122]. In diabetes, elevated oxidative stress plays a key role in the progression of complications and impairs the healing process of diabetic wounds [133]. Nuclear factor erythroid 2-related factor 2 (NRF2) effectively eliminates ROS and protects cells against oxidative stress by inducing the transcription of numerous antioxidants and cytoprotective genes through activation of the NRF2/antioxidant response element (ARE) complex. Therefore, EVs derived from ADSCs overexpressing NRF2 significantly improve diabetic wound healing by reducing the ulcerated area and inflammation, promoting cell proliferation, granulation tissue formation and angiogenesis, and upregulating growth factor expression while attenuating oxidative stress-related protein expression [123, 165]. In addition to protein-coding genes, microRNAs (miRNAs) are extensively employed for engineering ADSC-EVs to enhance their therapeutic activity. MiRNAs can be transfected as DNA constructs, overexpressed in ADSC cells and loaded into ADSC-EVs by the donor cells themselves [96], or directly electroporated into isolated ADSC-EVs as miRNA mimics [166]. Both strategies have been validated to yield positive outcomes in the treatment of chronic diabetic wounds. EVs secreted from miR-132-overexpressing ADSCs promote diabetic wound healing by upregulating angiogenesis, collagen deposition, and ECM fibronectin accumulation and attenuating local inflammation through nuclear factor-κB (NF-κB) signaling-mediated M2 macrophage polarization [96]. ADSC-EVs loaded with miR-21-5p via electroporation show similar accelerated healing rate in diabetes-associated wound models [166]. Circular RNAs (circRNAs), another type of noncoding RNA with significant biological functions, often act as sponges for miRNAs to prevent them from binding to their downstream target mRNAs [167]. This machinery has been utilized to enhance the therapeutic potential of ADSC-EVs. Circ_0001052-overexpressing ADSC-EVs promote angiogenesis in DFUs by sequestering miR-106a-5p, thereby disinhibiting the translation of its downstream target fibroblast growth factor-4 (FGF4), which is known to be involved in embryonic development, cell proliferation and differentiation [119]. As expected, published data have confirmed the improved therapeutic effects of these modified EVs on DFUs [119]. A similar approach has been reported for another circRNA, circ_0000250 [120]. The overexpression of circ_0000250 in ADSC-EVs results in enhanced healing in diabetic mice through the induction of autophagy mediated by the miR-128-3p/SIRT1 axis since epidermal SIRT1 is responsible for the modulation of inflammation, wound healing and cell migration [120]. In general, the application of bioengineering techniques to modify ADSC-EVs has demonstrated remarkable accessibility and potency in the treatment of chronic wounds associated with diabetes, thereby enabling robust and personalized therapy in future clinical settings.

In addition to their application in acute wounds, hydrogels and other scaffolds have also been combined with ADSC-EVs for localized and sustained release in treating chronic diabetic wounds. Hydrogels can serve as carriers for ADSC-EVs only, exerting therapeutic effects through the hypoxia-induced circ-Snhg11/miR-144-3p/NRF2/HIF-1α axis [117], which is identical by mechanism to that achieved by administering ADSC-EVs alone [116]. Alternatively, it can be dual-loaded with ADSC-EVs and an additional small chemical compound such as metformin to achieve synergistic efficacy [168]. Extensive efforts have been devoted to developing hydrogels into versatile platforms with multifunctional capabilities beyond mere EV delivery, including injectability, self-healing properties, antibacterial activity, and pH-responsive EV release, among others [169, 170]. These studies explicitly demonstrate significantly enhanced healing efficiency of multifunctional hydrogel-incorporated ADSC-EVs in full-thickness cutaneous wounds of diabetic models compared to those treated with either EVs or hydrogels alone [169, 170]. In addition to conventional hydrogels, alternative scaffolds, such as cell sheets and acellular fibers or membranes, have been applied as carriers of ADSC-EVs for combating chronic diabetic wounds. Studies have demonstrated that transplantation of ADSC sheets derived from rat epididymal adipose tissue enhances wound healing in diabetic rats [171]. Then, further studies have combined IRF1-overexpressing ADSC-EVs with ADSC sheets, resulting in further improvements in wound healing via the IRF1/miR-16-5p/SP5 axis [124]. Moreover, acellular amniotic membranes and functional micro/nanofibers composed of phosphoethanolamine phospholipid-grafted poly-L-lactic acid have been assessed as delivery platforms for ADSC-EVs in diabetic wound therapy. Both exhibit enhanced healing potential in diabetic models, characterized by augmented cell proliferation and migration, angiogenesis, collagen production and deposition, M2 macrophage polarization, and reduced inflammation, accompanied by the modulation of associated marker genes [172, 173]. As evidenced by these studies and similar ones, the continuous development and application of novel biomaterials will strongly facilitate the realization of ADSC-EV-based cell-free stem cell therapy.

### Chronic limb ischemia

Lower extremity peripheral arterial disease (PAD) can progress into chronic critical limb ischemia, which is characterized by intractable rest foot pain, gangrene, and tissue necrosis [174]. Chronic limb ischemia is a prevalent comorbidity in diabetic patients and frequently leads to chronic diabetic wounds (ulcers) and limb amputation due to inadequate tissue perfusion and dysfunctional revascularization [175].

**Fig. 3:**
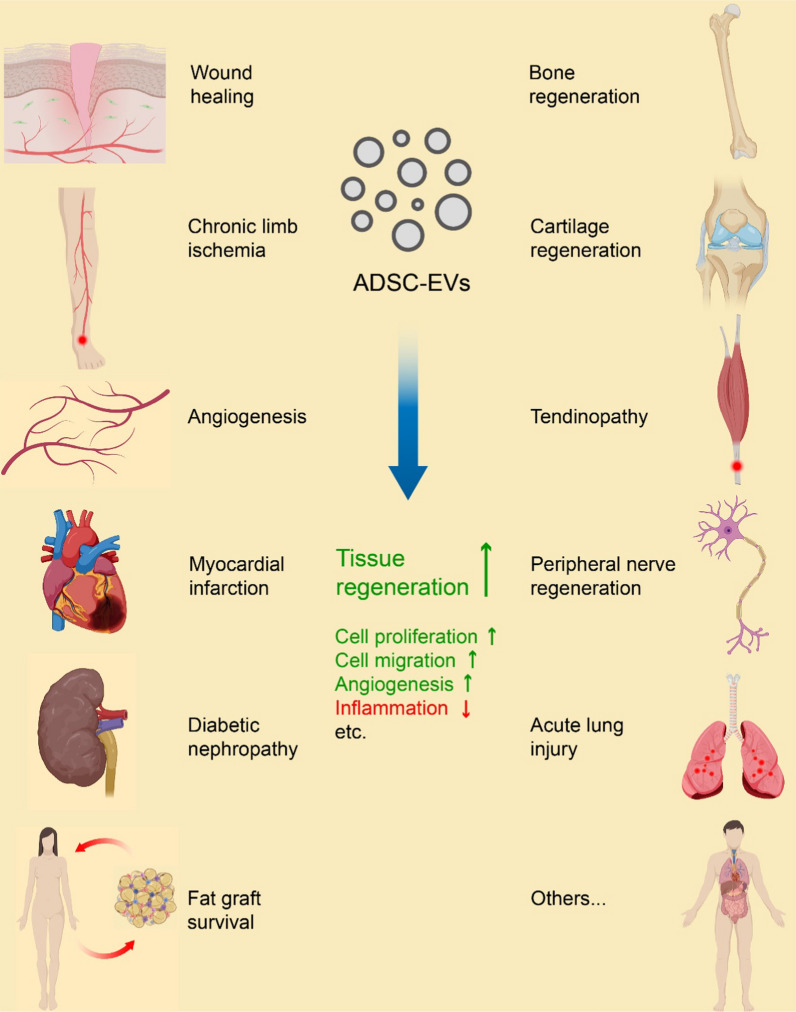
Regenerative medical conditions that are affected by ADSC-EVs and the major mechanisms of tissue regenerative efficacy. The recent advances in the regenerative medical use of ADSC-EVs focus mainly on these listed conditions. And, the most common primary mechanisms of tissue regenerative effects involve stimulated cell proliferation, migration and angiogenesis, and suppressed inflammation. Created with MedPeer (medpeer.cn)

The cornerstone of chronic limb ischemia treatment lies in revascularization or angiogenesis [174], which is supported by ADSC-EV administration (Fig. 2, 3). One study shows that ADSC-EVs promote cell proliferation, migration, and tube formation while reducing cell apoptosis in high glucose-conditioned HUVECs [125]. Additionally, they enhance blood perfusion, microvessel density, and muscle structural integrity in diabetic mice. The healing potential may be attributed to the upregulation of endothelial nitric oxide synthase (eNOS)/AKT/ERK/P-38 signaling pathways, along with the downregulation of activator protein-1 (AP-1)/ROS/NACHT-, leucine-rich repeat (LRR)- and pyrin domain (PYD)-containing protein 3 (NLRP3)/apoptosis-associated speck-like protein containing a CARD (ASC)/caspase-1/interleukin-1β (IL-1β) [125]. Furthermore, glyoxalase-1 (GLO-1) overexpression in ADSC-EVs further enhances their therapeutic potential [125]. Another study reveals that ADSC-EVs can polarize M1 macrophages toward an M2-like phenotype, characterized by reduced secretion of proinflammatory cytokines and increased production of proangiogenic factors [176]. This M2-like polarization promotes endothelial cell proliferation, migration, and tube formation. These effects have been verified in mouse hindlimb ischemia models with a Matrigel plug assay. Moreover, EVs from hypoxia-treated ADSCs exhibit even greater M2-like polarization and therapeutic potential [176]. Elevated levels of miR-21 and colony stimulating factor-1 (CSF-1)/CSF-1 receptor (CSF-1R) appear to play a pivotal role in initiating these beneficial effects [176]. These studies highlight the healing efficacy of ADSC-EVs on chronic limb ischemia while revealing their clinical potential for angiogenic therapy.

### Angiogenesis

Angiogenesis plays a critical role in wound healing and is essential for the treatment of ischemia and other tissue regeneration failures. Conventional cytokine-based angiogenic therapies utilizing growth factors such as vascular endothelial growth factors (VEGFs) and FGFs have demonstrated limited efficacy in clinical practice [177, 178]. Consequently, researchers have recently shifted their focus toward ADSC-based stem cell therapy and more advantageous ADSC-EV-based cell-free approach due to the ability of ADSCs to differentiate into endothelial cells or pericyte-like cells and to secrete proangiogenic growth factors and EVs [177].

Extensive efforts have been made to elucidate the correlation between angiogenesis and ADSC-EV administration, as well as the underlying mechanisms involved. Numerous studies have validated the positive impact of ADSC-EVs on angiogenesis [94, 98, 99, 101, 179–181] (Fig. 2, 3). Specifically, ADSC-EVs have been demonstrated to enhance endothelial cell proliferation, migration, and tube formation, as evidenced by increased vessel length and number of junctions and branches. These effects are attributed to the upregulation of growth factors and receptors such as VEGFs and their receptors, epidermal growth factor (EGF), platelet-derived growth factors (PDGFs), and FGFs, as well as proliferation markers, including cyclin proteins. In vivo studies in rodents have consistently shown improved neovascularization or angiogenesis along with accelerated wound closure due to enhanced re-epithelialization and collagen deposition. The therapeutic effects are mediated through exosomal miRNA-initiated signaling pathways involving axes of the miR-486-5p/SP5/cyclin D2 [94], the miR-126/sprouty-related EVH1 domain containing 1 (SPRED1)/ERK1/2 [98], the miR-125a/Drosophila delta-like 4 homolog (DLL4) [99], and the miR-31/factor-inhibiting HIF-1 (FIH1)/HIF-1α [101]. Interestingly, both hypoxia and metabolic conditions of ADSCs have been shown to influence the proangiogenic potential of derived EVs [98, 181]. Notably, compared with normoxic ADSC-EVs, hypoxic ADSC-EVs exhibit enhanced proliferation, migration and tube formation in HUVECs and improved neovascularization in a mouse model [181]. These therapeutic benefits are attributed to increased levels of growth factors and receptors, such as VEGF, EGF, FGF, VEGF-R2, and VEGF-R3, and chemokines, including monocyte chemotactic protein 2 (MCP-2) and MCP-4 [181]. However, obesity or hypertrophy can impair the proangiogenic potential of ADSC-EVs. Compared with those from lean individuals, EVs derived from ADSCs isolated from individuals with obesity exhibit attenuated potential for inducing endothelial cell proliferation, migration, and overall angiogenesis [98]. This impairment is associated with a reduction in exosomal miR-126 [98]. Moreover, treatment with palmitic acid induces ADSCs to generate EVs that recapitulate the cargo of obesity-associated ADSC-EVs. Additionally, high glucose-treatment further decreases the miR-126 level enclosed in ADSC-EVs, along with attenuated angiogenic potential [98]. Note that the angiogenic potential of ADSC-EVs is influenced not only by the composition of the culture media but also by the type of cell culture. It has been reported that EVs derived from 3D-spheroid-cultured ADSCs exhibit superior potency in promoting angiogenesis in HUVECs compared to those obtained from 2D monolayer-cultured cells [180]. Because the 3D-culturing resembles the natural local microenvironment of tissue in vivo, hopefully EVs directly extracted from adipose tissue may display higher regenerative potency [182]. Reported data have demonstrated the therapeutic efficacy of adipose tissue-derived EVs (AT-EVs) [183, 184]. However, AT-EVs cannot be strictly termed as ADSC-EVs, since the adipose tissue is composed of multiple cell types including ADSCs and others such as mature adipocytes and macrophages. And, ADSCs make up only around 12–18% but the most regeneratively active fraction of the total adipose cell population [10]. Therefore, further investigation is needed to compare the therapeutic potency of these two kinds of EVs. In conclusion, recent research has highlighted the potential of ADSC-EVs for future clinical applications in treating diseases related to ischemia and angiogenic failure, which are the leading causes of limb disability, necrosis, and amputation.

### Myocardial infarction

Myocardial infarction (MI), commonly referred to as heart attack, is a severe medical condition characterized by the damage or death of myocardial tissue due to prolonged blockage of blood flow to that specific area of heart [185]. The pathology underlying this occlusion involves atherosclerosis, an inflammatory process leading to the narrowing and hardening of arteries with the buildup of plaque, thereby impeding blood circulation [186]. This progression arises from chronic inflammation and manifests as the accumulation of cholesterol, lipids, and other substances on the vascular wall [187]. Plaque rupture triggers clot formation, obstructing blood supply to cardiac muscle. Immediate management strategies for MI aim at promptly restoring blood flow through invasive procedures such as percutaneous coronary intervention (PCI) and coronary artery bypass grafting (CABG) or pharmacological interventions. Long-term management encompasses lifestyle modifications and medications targeting risk reduction for future cardiovascular events while promoting tissue repair following MI damage; herein lies the potential role of ADSC-EVs.

According to the available references, the therapeutic potential of ADSC-EVs has been demonstrated in ameliorating MI injury by promoting cardiac angiogenesis, inhibiting cardiomyocyte apoptosis, and improving myocardial function [102, 188, 189] (Fig. 3). ADSC-EVs have shown efficacy in reducing obesity, enhancing insulin sensitivity, and maintaining metabolic homeostasis in obese mice, indicating their potential for alleviating metabolic disorders associated with MI [137]. Moreover, the aforementioned promotion of wound healing and vascularization by ADSC-EVs suggests their potential for addressing tissue damage related to MI [123]. Various signaling pathways are involved in mediating these therapeutic effects. It has been reported that ADSC-EVs promote cardiac regeneration and inhibit post-MI adverse remodeling through activation of the PI3K/AKT pathway. The PI3K/STAT3 pathway has also been implicated in modulating macrophage polarization to ameliorate cardiac fibrosis in infarcted hearts [190]. Additionally, EV-encapsulated miR-205, miR-196a-5p, miR-425-5p, and miR-31 (via the miR-31/FIH1/HIF-1α axis) have been identified as crucial initiators of cardiac angiogenic pathways underlying the attenuation of MI injury and the promotion of cardiac function recovery by ADSC-EVs [102, 188, 189]. Furthermore, miR-146a-overexpressing ADSC-EVs effectively attenuate acute MI-induced myocardial damage through downregulation of early growth response factor 1 (EGR1) and subsequent reversal of Toll-like receptor 4 (TLR4)/NF-κB signal activation [191, 192]. Collectively, these studies suggest that ADSC-EVs could be a valuable therapeutic tool for addressing the aftermath of MI, offering potential benefits in promoting cardiac regeneration, attenuating MI injury, and improving myocardial function.

### Diabetic nephropathy

Diabetic nephropathy is a severe complication of diabetes characterized by persistent albuminuria, a decrease in renal function, and increased cardiovascular morbidity and mortality [193]. It is the primary cause of chronic kidney disease in patients who are receiving renal replacement therapy [193]. The incidence of diabetic nephropathy is increasing concomitantly with the global surge in cases of diabetes mellitus [194].

Recent investigations have revealed that ADSC-EVs can mitigate pathological symptoms such as increased urine protein levels, serum creatinine (Scr) levels, blood urea nitrogen (BUN) levels, and podocyte apoptosis. This is achieved through the promotion of autophagy flux, reduction of inflammation, and attenuation of ROS levels [103, 105, 195]. The underlying signaling pathways responsible for these effects have been identified as the Kelch-like ECH-associated protein 1 (KEAP1)/NRF2/family with sequence similarity 129, member B (FAM129B) pathway [195] and the exosomal miR-26a-5p/TLR4/NF-κB pathway [105], leading to inflammatory inhibition. Additionally, the exosomal miR-486/SMAD1/mammalian target of rapamycin (mTOR) pathway [103] functions to activate autophagy. In conclusion, the therapeutic potential of ADSC-EVs in diabetic nephropathy is promising as a novel therapeutic strategy (Fig. 3).

### Fat graft survival

Autologous fat grafting is a procedure in which adipose tissue is removed from one anatomical site and subsequently transplanted to another area within the same individual, with the aim of augmenting or restoring soft tissue volume [196]. This technique has garnered considerable attention in both aesthetic and reconstructive medicine due to its extensive applicability in facial and hand rejuvenation, breast augmentation and reconstruction, and body contouring [197–199]. Nevertheless, the primary obstacle impeding further advance and widespread adoption of fat grafting lies in the unpredictable resorption rates and low survival rates of the transplanted fat [197]. These challenges are believed to stem primarily from inadequate neovascularization post-transplantation [199]. Addressing these issues is crucial for enhancing the predictability and success rates of autologous fat grafting.

ADSCs have been demonstrated to play a regenerative role and facilitate the proliferation, migration, and secretion of fibroblasts and keratinocytes, thereby contributing to the replenishment and augmentation of soft tissues [200]. Co-transplantation of autologous adipose tissue with human ADSCs has been shown to enhance fat graft retention by promoting neovascularization and angiogenesis primarily through paracrine mechanisms [201, 202]. Considering that ADSC-EVs retain the therapeutic properties of ADSCs while offering certain advantages over live cells, as previously mentioned, researchers have investigated the effects of administering ADSC-EVs in fat grafting procedures and accumulated evidence supporting their significant involvement in promoting fat graft survival and adipose tissue regeneration (Fig. 3). When introduced into fat grafts, these EVs are capable of increasing neovascularization and graft retention, inhibiting fibrosis and necrosis, and inducing M2 polarization while suppressing M1 polarization and graft inflammation [198]. Further studies have indicated that ADSC-EVs can enhance fat graft retention rates by stimulating endothelial cell angiogenesis and facilitating ADSC adipogenesis through enhanced cell proliferation and migration, along with modulating the expression of marker genes [197, 199, 203, 204]. The underlying mechanisms responsible for these effects involve the modulation of various signaling pathways, such as the Wnt/β-catenin pathway [197, 204]. These findings underscore the potential utility of ADSC-EVs as a promising strategy for enhancing fat graft survival and adipose tissue regeneration.

### Bone regeneration

The skeletal system plays a fundamental role in providing structural support, safeguarding vital organs, and facilitating movement. Bone regeneration and repair are indispensable for maintaining skeletal integrity, mobility, and overall physiological function following traumatic injuries, degenerative diseases or surgical interventions. This intricate biological process involves numerous cellular and molecular events that are critical for addressing medical conditions such as fractures, osteoporosis, bone defects, and orthopedic surgeries [205].

According to recent publications, researchers have demonstrated the crucial role of ADSC-EVs in promoting bone regeneration (Fig. 3). ADSC-EVs activate the proliferation, migration and osteogenesis of bone marrow-derived mesenchymal stem cells (BMSCs), stimulate angiogenesis in HUVECs, and induce M2 polarization while inhibiting the osteoclastogenesis of RAW264.7 macrophages [107, 206, 207]. Enhanced bone formation in animal models of bone defects has validated the osteogenic effect of these EVs [106, 107, 206]. The therapeutic potential of ADSC-EVs in bone regeneration and repair is mediated via various signaling pathways. One critical pathway that plays a key role in the promotion of osteogenesis by ADSC-EVs is the PI3K/AKT pathway [164, 190], leading to improved osteogenic effects and bone regeneration [208]. Furthermore, ADSC-EV-enclosed cytokines such as osteoprotegerin (OPG) and miRNAs such as miR-21-5p and let-7b-5p have been reported to hamper osteoclastogenesis by downregulating genes associated with bone resorption, suggesting the potential therapeutic use of ADSC-EVs in the treatment of osteoporosis [107]. To effectively utilize ADSC-EVs for bone regeneration, several application strategies have been proposed. These include genetic modifications of ADSCs to enhance the potency of derived EVs [209], engineering of the membrane protein of ADSC-EVs to improve their homing and retention capacities [206], and the use of biomaterial scaffolds or hydrogels to help immobilize the EVs and introduce extra osteoinductive factors to the healing site [207, 208, 210, 211]. The integration of different engineering strategies to maximize the bone regenerative efficiency of ADSC-EVs has also been evaluated. The hydrogel-encapsulated ADSC-EVs with overexpressed miR-375 enhance bone regenerative capacity with a slow and controlled release in a rat model of bone defect [106]. Challenges in the application of ADSC-EVs for bone regeneration may include the need for understanding the interactions between ADSC-EVs and the host environment, standardization of procedures for their use, and further investigations to optimize their effectiveness and delivery methods. In general, these findings support the significant therapeutic potential of ADSC-EVs in bone regeneration and repair and emphasize the need for future research to fully elucidate the underlying mechanisms and optimize their clinical application.

### Cartilage regeneration

The significance of cartilage lies in its pivotal role in maintaining joint health and mobility and the subsequent impacts on overall quality of life. Articular cartilage serves as a crucial structural component within the skeletal system, providing a smooth and lubricated surface for joint movement and load distribution. Therefore, the regeneration and repair of articular cartilage are imperative for preventing the progression of osteoarthritis, which affects a substantial number of individuals, along with other joint-related disorders [212].

Multiple studies have demonstrated the capacity of ADSC-EVs to promote the proliferation, migration, chondrogenic differentiation, and osteogenic differentiation of BMSCs [213, 214]. Compared to bone marrow- and synovium-derived MSC-EVs, adipose-derived MSC-EVs display superior regenerative efficacy for cartilage and bone in a mouse model [213]. The therapeutic potential of ADSC-EVs has been assessed in the context of not only osteochondral regeneration but also osteoarthritis. In both in vitro and in vivo models of inflammatory osteoarthritis, ADSC-EVs are found to stimulate chondrogenesis while reducing cartilage degeneration and suppressing inflammation. Consistent modulation of marker genes are also verified associating with chondrocyte viability, cartilage matrix homeostasis, macrophage polarization, and inflammatory suppression [214–216]. The activation of specific signaling pathways, such as the Wnt/β-catenin, PI3K/AKT, the adenosine 5’-monophosphate (AMP)-activated protein kinase (AMPK), and the MAPK-ERK/1/2 pathways, has been implicated in mediating these therapeutic effects [213, 216]. Supported by an increasing body of evidence, the ability of ADSC-EVs to promote chondrogenesis while decreasing cartilage lesions and inhibiting inflammation holds promise for the development of innovative regenerative therapies for cartilage-related disorders (Fig. 3).

### Tendinopathy and tendon healing

Tendinopathy is a prevalent musculoskeletal disorder characterized by a dysregulated collagen matrix, chronic low-grade inflammation and tissue degeneration, resulting in activity-related chronic pain and functional decline [217]. The etiology of tendinopathy is multifactorial, involving extrinsic conditions such as trauma and chronic overuse, as well as intrinsic conditions such as obesity, inflammation, and genetics [217, 218]. It poses a significant burden on healthcare systems and represents a common cause of pain and disability among athletes and sedentary individuals [219]. The limited regenerative capacity of tendons, in conjunction with the intricate pathology of tendinopathy, emphasizes the criticality of understanding the underlying mechanisms and developing efficacious therapeutic strategies for treating tendon injuries and disorders.

Recent studies have highlighted the therapeutic potential of ADSC-EVs in tissue regeneration, specifically in the healing of traumatized Achilles tendons [220, 221]. The promotion of Achilles tendon regeneration and repair by ADSC-EVs is characterized by enhanced proliferation and migration of tendon stem cells, along with increased viability, reduced senescence, and elevated collagen production in tenocytes. These effects are mediated through specific signaling pathways, including the SMAD1/5/9 and SMAD2/3 pathways [222], as well as the nicotinamide phosphoribosyltransferase (NAMPT)/SIRT1/peroxisome proliferator activated receptor γ (PPARγ)/PPARγ coactivator 1-α (PGC1-α) pathway [127]. Furthermore, ADSC-EVs exhibit anti-inflammatory effects by suppressing M1 macrophage polarization while stimulating M2 macrophage polarization. This dual action makes them promising therapeutic agents for promoting both tendon regeneration and inflammatory suppression in Achilles tendinopathy [104, 127, 222]. Moreover, the potential of ADSC-EVs for rotator cuff tendon regeneration and repair has been evaluated [223–226]. Administration of ADSC-EVs has been shown to enhance collagen deposition, promote tendon maturation and myofiber regeneration, improve histological and biomechanical properties, and reduce tendon degeneration, atrophy, and fatty infiltration in animal models of supraspinatus and infraspinatus tendon injury [223–225]. Similarly, immunomodulation characterized by augmented M2 polarization and attenuated M1 polarization has also been observed during these therapeutic interventions [223, 226]. In summary, there is substantial evidence supporting the therapeutic potential of ADSC-EVs in tendinopathy and tendon healing (Fig. 3). These studies collectively demonstrate their potential for improving regenerative capacity while mitigating inflammation in both the Achilles and rotator cuff tendons, thereby highlighting their significant promise as a therapeutic agent within the field of regenerative medicine.

### Peripheral nerve regeneration

The peripheral nervous system is responsible for transmitting signals between the central nervous system and the rest of the body. Injuries to peripheral nerves can arise from various forms of damage or trauma, leading to a range of debilitating symptoms, such as sensory loss, muscle weakness, numbness, tingling, and chronic pain characterized by sharp, burning, or throbbing sensations. These impairments significantly impact an individual’s ability to carry out daily activities and may result in long-term disability [227, 228]. Consequently, promoting effective peripheral nerve regeneration holds immense medical significance in addressing these challenges and enhancing patient outcomes.

Recently, research has demonstrated the endocytosis-mediated internalization of ADSC-EVs by Schwann cells, which originate from the neural crest and play a primary role in axonal myelination within the peripheral nervous system [229, 230]. Myelination is essential for facilitating rapid nerve impulse conduction [229]. Numerous studies have highlighted the therapeutic potential of ADSC-EVs in promoting peripheral nerve regeneration post-injury through various mechanisms, such as cell proliferation, migration, myelination, neurotrophic factor secretion, and autophagy induction in Schwann cells [109, 230–232]. Consistently, animal experiments administering ADSC-EVs to rats with sciatic nerve injuries have shown enhanced regeneration of both the myelin sheath and axons and improved restoration of denervated muscle atrophy via optimized Schwann cell function [109, 231, 232]. Additionally, ADSC-EVs have been demonstrated to facilitate the recovery of erectile function following cavernous nerve injury, further emphasizing their potential application in treating peripheral nerve injuries [233]. Furthermore, ADSC-EV-encapsulated miRNA is found to promote Schwann cell proliferation and migration via the miR-22-3p/phosphatase and tensin homolog deleted on chromosome 10 (PTEN)/AKT/mTOR axis [110]. Another ADSC-EV-miRNA is reported to induce Schwann cell autophagy through the miR-26b/karyopherin subunit α-2 (KPNA2) axis [109]. These investigations elucidate the underlying molecular machinery governing the efficacy of ADSC-EVs in peripheral nerve regeneration. Overall, these findings provide valuable insights into the role of ADSC-EVs in promoting peripheral nerve regeneration and functional recovery while supporting their potential clinical application in treating peripheral nerve injuries (Fig. 3).

### Acute lung injury

Acute lung injury (ALI), also known as acute respiratory distress syndrome (ARDS) in its severe form, is a prevalent and life-threatening pulmonary disease characterized by acute alveolar damage, pronounced inflammation, heightened vascular permeability, and substantial protein-rich pulmonary edema, leading to reduced lung compliance, impaired gas exchange, hypoxemia and respiratory failure [234, 235]. The pathogenesis of ALI primarily involves disruption of the lung endothelial and epithelial barriers induced by acute inflammation [234]. ADSC-EVs have been demonstrated to ameliorate ALI through mechanisms that include immunomodulation of alveolar macrophages and protection of the pulmonary endothelial barrier. In the recent pandemic of coronavirus disease 2019 (COVID-19), approximately 15–30% of people hospitalized with COVID-19 will develop COVID-19 associated ARDS (CARDS) [236, 237]. The therapeutic effects of MSC-EVs including ADSC-EVs in COVID-19 are currently being assessed with dozens of clinical trials (Table 1).

It has been demonstrated that the administration of ADSC-EVs in alveolar macrophages results in decreased secretion of inflammatory cytokines and increased production of anti-inflammatory cytokines [19, 238]. And, the transfer of mitochondrial components derived from donor cells through EVs plays a significant role in mediating these therapeutic effects when internalized by recipient alveolar macrophages. This internalization leads to an increase in mtDNA levels, mitochondrial membrane potential, mitochondrial oxidative phosphorylation (OXPHOS) activity, and ATP generation while relieving mitochondrial ROS stress [19]. In vivo assessments in mice with cecal ligation and puncture (CLP)-induced ALI have consistently revealed anti-inflammatory outcomes, such as reduced macrophage aggregation, downregulated pro-inflammatory cytokines, and alleviated pulmonary edema and vascular leakage, which are associated with improved survival rates [238]. In addition, the administration of ADSC-EVs has been shown to facilitate recovery from pulmonary microvascular endothelial cell (PMVEC) injury. These EVs effectively inhibit excessive inflammatory response-induced ROS accumulation; cell damage, including apoptosis and ferroptosis; tight junction damage; and high permeability of PMVECs [111, 239]. Furthermore, ADSC-EVs protect against cigarette smoke-induced chronic obstructive pulmonary disease (COPD) [240] and silicosis [241] by regulating macrophages and suppressing inflammation. Moreover, they have shown efficacy in treating ventilator-induced lung injury (VILI) by repairing the pulmonary endothelial barrier and ameliorating the inflammatory response [242]. In conclusion, these findings strongly support the immunomodulatory and pulmonary endothelial cell protective properties of ADSC-EVs in the context of ALI and related conditions (Fig. 3). These findings underscore the potential therapeutic efficacy of ADSC-EVs as a promising intervention for these diseases, necessitating further investigations to understand the underlying mechanisms and optimize strategies for future clinical application.

### Other regenerative medical conditions affected by ADSC-EVs

In addition to their effects on the aforementioned diseases, ADSC-EVs have also displayed therapeutic potential in various medical conditions that require tissue regeneration and immunomodulation (Fig. 3). According to the latest literature available to the authors, ADSC-EVs ameliorate hepatic ischemia–reperfusion injury [243], skeletal muscle injury [126, 244], diabetic [245, 246] or non-diabetic [247] erectile dysfunction, and thin endometrium-induced infertility [248], primarily through the activation of tissue regeneration characterized by enhanced cell proliferation, differentiation, angiogenesis, reduced apoptosis, and modulated production of cell factors regulating these processes. Additionally, ADSC-EV-mediated inflammatory suppression has been reported as a major underlying mechanism for treating atopic dermatitis [249, 250], sepsis [251, 252], and atherosclerosis-induced vascular lesions [253], along with other conditions of immunosuppression failure listed in this review. In these contexts, ADSC-EVs downregulate the expression of inflammatory cytokines while decreasing immunocyte infiltration and reducing ROS accumulation. They also inhibit macrophage M1 polarization while stimulating M2 polarization [249–253]. Furthermore, ADSC-EVs exhibit antifibrotic effects on hepatic fibrosis by reducing fibrotic collagen deposition and liver inflammation while restoring liver function [112, 254, 255]. Moreover, ADSC-EVs have been found to protect against metabolic disturbance and alleviate polycystic ovary syndrome by maintaining liver metabolic homeostasis [108]. Collectively, these studies highlight the versatile applications of ADSC-EVs in promoting tissue regeneration and reducing inflammation and fibrosis while maintaining metabolic homeostasis, thus strongly supporting their role as a promising therapeutic strategy in regenerative medicine.

### Conclusions and perspectives

ADSCs have gained recognition for their significant potential in regenerative medicine due to their ability to differentiate into various cell lineages and exert autocrine/paracrine effects through the secretion of growth factors, cytokines, chemokines, miRNAs, proteins, and diverse important mediators enclosed in EVs [75]. Utilizing ADSC-derived EVs offers several advantages, including ease of transportation and storage, low immunogenicity, and no potential tumorigenicity, while preserving the therapeutic activity of donor ADSCs [200]. The reviewed studies have demonstrated that ADSC-EVs represent a novel therapeutic approach in the field of regenerative medicine due to their pivotal roles in various biological processes, such as cell proliferation, migration, angiogenesis, apoptosis, tissue regeneration, and immunomodulation [78]. They have been applied to the afore-listed regenerative medical conditions and have shown the most promising outcomes in skin wounds, scars, bone injuries, and fat grafting associated with plastic and cosmetic surgery. Utilizing adipose tissue as the source of MSCs provides numerous advantages, including abundant clinical source availability, resulting high EV yields, and easier and less invasive surgical technique to obtain the adipose tissue compared to other tissue sources, which is uniquely convenient for plastic surgeons and researchers since plastic surgeons frequently perform liposuction and autologous fat transplantation [76].

However, several notable challenges still need to be addressed before the clinical application of ADSC-EVs can be realized. Currently, the clinical translation of ADSC-EVs is hindered by limitations such as insufficient understanding of the mechanisms and interactions between ADSC-EVs and the host environments; variations in EV contents and bioactivities across tissues, individuals and their metabolic states; less-optimized therapeutic efficiency and delivery methods; an absence of standardized protocols and automatic workflows specific for adipose tissue and ADSC isolation, large-scale production, transport and preservation; a lack of quality control for EV sources; and the inadequate standards for the characterization of EV products [75, 79, 256]. Please note that adipose tissue is dynamically and deeply associated with and regulating the systemic metabolic homeostasis [1], which could potentially augment the variation of EV contents across individuals and their metabolic states. For example, ADSC-EVs collected from normal individuals and individuals with obesity might show inconsistent efficacies [98]. As a result, further studies, engineering optimizations, and the establishment of these procedures and standards, with a specific consideration of donor metabolic states, will be essential for facilitating the transition of ADSC-EV therapy to clinical practice [256]. Overall, the therapeutic potential of ADSC-EVs in tissue regeneration is strongly supported by their pro-proliferative, proangiogenic, regenerative, and immunomodulatory properties. The future clinical applications of these EVs hold great promise for addressing diverse medical conditions and advancing regenerative medicine.

## Data Availability

No datasets were generated or analysed during the current study.

## Abbreviations

ACVR2A: Activin A receptor Type 2A
ADSC: Adipose-derived stem cell
ADSC-EV: Adipose-derived stem cell (ADSC)-derived extracellular vesicle (EV)
ALI: Acute lung injury
AMPK: Adenosine 5’-monophosphate (AMP)-activated protein kinase
AP-1: Activator protein-1
ARDS: Acute respiratory distress syndrome
ARE: Antioxidant response element
ARRDC1: Arrestin domain-containing protein 1
ASC: Apoptosis-associated speck-like protein containing a CARD
α-SMA: α-Smooth muscle actin
AT-EV: Adipose tissue-derived extracellular vesicle (EV)
BMSC: Bone marrow-derived mesenchymal stem cell
BTG2: B-cell translocation gene 2
BUN: Blood urea nitrogen
CABG: Coronary artery bypass grafting
CARDS: COVID-19 associated acute respiratory distress syndrome (ARDS)
CCL1: C–C motif chemokine ligand 1
CircRNA: Circular RNA
CLP: Cecal ligation and puncture
COPD: Chronic obstructive pulmonary disease
COVID-19: Coronavirus disease 2019
CSF-1: Colony stimulating factor-1
CSF-1R: Colony stimulating factor (CSF)-1 receptor
DFU: Diabetic foot ulcer
DLL4: Drosophila delta-like 4 homolog
ECM: Extracellular matrix
EGF: Epidermal growth factor
EGFR: Epidermal growth factor receptor
EGR1: Early growth response factor 1
eNOS: Endothelial Nitric oxide synthase
ERK: Extracellular signal-regulated kinase
ESCRT: Endosomal sorting complex required for transport
EV: Extracellular vesicle
FAM129B: Family with sequence similarity 129, member B
FasL: Fas ligand
FGF: Fibroblast growth factor
FIH1: Factor-inhibiting hypoxia-inducible factor (HIF)-1
GLO-1: Glyoxalase-1
HaCaT cells: Cultured human keratinocytes
HDF: Human dermal fibroblast
HIF-1α: Hypoxia-inducible factor-1α
HSF: Hypertrophic scar fibroblast
HUVEC: Human umbilical vein endothelial cell
IL-1β: Interleukin-1β
IL-17RA: Interleukin-17 receptor A
IRS1: Insulin receptor substrate 1
KEAP1: Kelch-like ECH-associated protein 1
KF: Keloid fibroblast
KPNA2: Karyopherin subunit α-2
MCP: Monocyte chemotactic protein
MI: Myocardial infarction
miRNA: MicroRNA
MMP: Matrix metalloproteinase
MSC: Mesenchymal stem cell
MSC-EV: Mesenchymal stem cell (MSC)-derived extracellular vesicle (EV)
mTOR: Mammalian Target of rapamycin
MVB: Multivesicular body
NAMPT: Nicotinamide phosphoribosyltransferase
NF-κB: Nuclear factor-κB
NLRP3: NACHT-, leucine-rich repeat (LRR)- and pyrin domain (PYD)-containing protein 3
NRF2: Nuclear factor erythroid 2-related factor 2
NRG1: Neuregulin 1
OPG: Osteoprotegerin
OXPHOS: Oxidative phosphorylation
P38MAPK: P38 mitogen activated protein kinase
PAD: Peripheral arterial disease
PAQR3: Progestin and adipoQ receptor family member 3
PCI: Percutaneous coronary intervention
PDGF: Platelet-derived growth factor
PGC1-α: PPARγ coactivator 1-α
PI3K: Phosphatidylinositol 3-kinase
PIK3R2: Phosphoinositide-3-kinase regulatory subunit 2
PMVEC: Pulmonary microvascular endothelial cell
PPARγ: Peroxisome proliferator activated receptor γ
PTEN: Phosphatase and tensin homolog deleted on chromosome 10
ROS: Reactive oxygen species
RUNX3: RUNX family transcription factor 3
Scr: Serum creatinine
SIP1: Small mothers against decapentaplegic protein (SMAD)-interacting protein-1
SIRT3: Sirtuin 3
SMAD: Small mothers against decapentaplegic protein
SOD2: Superoxide dismutase 2
SPRED1: Sprouty-related EVH1 domain containing 1
SUFU: SUFU negative regulator of Hedgehog signaling
SVF: Stromal vascular fraction
T2D: Type 2 diabetes
TGF-β: Transforming growth factor-β
TIMP: Tissue inhibitor of metalloproteinases
TLR4: Toll-like receptor 4
TSG101: Tumor susceptibility gene 101
TSPAN: Tetraspanin
TWIST1: Twist basic helix-loop-helix transcription factor 1
UVB: Ultraviolet B
VEGF: Vascular endothelial growth factor
VILI: Ventilator-induced lung injury
VPS4: Vacuolar protein sorting-associated protein 4
